# SUPPORT MY WAY: Supporting Young People After Treatment for Cancer: What Is Needed, When This Is Needed and How This Can Be Best Delivered

**DOI:** 10.3390/curroncol32060361

**Published:** 2025-06-19

**Authors:** Nicole Collaço, Charlotte Ralph, Peter Dawes, Anne-Sophie Darlington, Andrew Davies, Ramya Ramanujachar, Louise Hooker, Samantha Sodergren

**Affiliations:** 1 School of Health Sciences, University of Southampton, Southampton SO17 1BJ, UK; nicole.collaco@nhs.net (N.C.); pd1n22@soton.ac.uk (P.D.); a.darlington@soton.ac.uk (A.-S.D.); 2 Patient Partner; 3 School of Cancer Sciences, University of Southampton, Southampton SO17 1BJ, UK; a.davies@soton.ac.uk; 4 University Hospital Southampton, Southampton SO16 6YD, UK; ramya.ramanujachar@uhs.nhs.uk (R.R.); louise.hooker@uhs.nhs.uk (L.H.)

**Keywords:** post treatment support, teenagers and young adults, cancer, oncology, cancer survivorship

## Abstract

As survival rates for teenagers and young adults (TYAs) with cancer exceed 80%, they are living longer post treatment, yet often experience prolonged health and quality of life concerns. Many TYAs also experience unmet support needs. This study aimed to identify TYAs support needs following treatment at a UK hospital and explore how and when TYAs prefer to receive support. This study involved two phases: Phase 1 involved semi-structured interviews with 16 TYAs, 1–6 years post-treatment, aged 16–25 years at time of treatment completion and examined their experiences of support services, and preferences for future care. Phase 2 consisted of co-design workshops with eight TYAs and feedback from five healthcare/allied professionals (HCAPs) to refine and develop recommendations. Phase 1 findings revealed six key themes: (1) survivorship as disrupted continuity; (2) negotiating legitimacy and relational safety in help seeking; (3) support offered vs. support sought: pathways of referral and self-initiation; (4) emotional readiness as context dependent and non-linear; (5) support as an ecosystem, not a moment; and (6) personalised autonomy in support engagement. Phase 2 findings informed recommendations that emphasise the importance of flexible, personalised, and accessible post-treatment support, with pathways of care/support that can adapt to TYAs changing needs and preferences over time.

## Simple Summary

A diagnosis of cancer is distressing at any age, but for teenagers and young adults the effects are likely to be more profound and extend far beyond the end of treatment. Recognising and addressing these long-term impacts is crucial. The aims of this study were firstly to identify the support needs of teenagers and young adults aged 16–25 who had completed cancer treatment 1–6 years earlier, and secondly to create recommendations for future practice. We asked 16 teenagers and young adults to share their experiences and then conducted workshops with 8 teenagers and young adults and asked five healthcare professionals to develop recommendations for improved support. Teenagers and young adults told us that the end of treatment was disruptive for them, they felt uncertain about seeking help with the offering of support not always matched to their needs. Support needs change over time with preferences for ongoing, personalised support. Flexible and accessible support services were recommended. The findings highlight the importance of developing support systems suitable for the changing needs of teenagers and young adults, ensuring they receive the care and resources necessary for their long-term wellbeing.

## 1. Introduction

The number of young people aged 15–24 years, often referred to as teenagers and young adults (TYAs), diagnosed with cancer each year in the United Kingdom (UK) is estimated to be 2300, accounting for less than 1% of all new cancer diagnoses. However, incidence has increased since the turn of the century [1]. With advancements in treatment, more than 87% of TYAs diagnosed with cancer in the UK now survive for at least five years although some cancer types have much lower survival rates [1]. Despite this progress, improved outcomes for TYAs fall behind those reported for children with cancer [2].

Attention to quality of life after cancer is important, as TYAs are likely to spend a considerable part of their lives as a “survivor” of cancer, with its treatment often leaving a lasting impact. Long-term sequelae can include a heightened risk of certain health conditions and damage to vital organs and bodily systems, for example, cardiovascular, endocrine, urinary, immune, and gastrointestinal systems, as well as causing potential fertility problems [3]. In addition, the psychosocial consequences of managing these late effects coupled with the reality of cancer becoming part of their personal narrative can be profound. TYAs are at a pivotal developmental stage, navigating identity, seeking autonomy, completing education, career development and building romantic relationships [4]. It is often only after treatment ends that TYAs begin to fully process the physical and psychological impact of their experience. TYAs often describe feeling ill-equipped for life beyond treatment and experience fear and uncertainty when the “safety bubble” of treatment and regular contact with the healthcare team cease to exist [5].

Given their diverse needs, providing adequate follow-up care for TYAs is complex. Existing support models often fall short due to a lack of standardised, tailored guidelines. These models also tend to overlook the unique medical, psychosocial, and care coordination needs of TYAs, which limits the effectiveness of follow-up care [6]. While international guidelines exist for TYA survivorship care, their implementation varies widely [7]. Shared care between oncology and primary care providers is considered the ideal model, but it may not be feasible in practice settings with weak primary care infrastructure [8,9]. One notable example of a successful survivorship model is The Princess Margaret Adolescent and Young Adult (AYA) Program in Canada [10]. A Clinical Nurse Specialist (CNS) supports patients (aged <39) through tailored referrals. The programme addresses medical and psychosocial issues, including fertility, nutrition, sexual health, and return to work/school. An evaluation of the programme [11] demonstrated the benefits of this structured, multidisciplinary survivorship care.

Drawing on research within childhood cancer survivors suggests that those attending long-term follow-up (LTFU) programmes generally experience better long-term physical and psychosocial health outcomes compared to those who do not [12]. However, these programmes are often designed without specific input from TYAs, potentially limiting their effectiveness for this distinct population. Furthermore, many experience barriers to participation, such as limited awareness about risks or late effects, fear of transitioning from oncology providers, and financial or geographical constraints in accessing care [13,14]. Additionally, primary healthcare professionals (HCPs) may lack familiarity and confidence with cancer related-risks and recommended health surveillance, while the complexity of the healthcare system further challenges LTFU care delivery [15,16,17].

In the UK, healthcare is publicly funded through the National Health Service (NHS), which is divided into primary care (e.g., general practitioners) and specialist care (e.g., hospitals and medical specialists). The NHS specification for TYA cancer services requires the TYA principal treatment centre to offer a comprehensive long-term follow-up package for cancer survivors, including psychosocial support and rapid re-access when required [18]. However, significant differences between paediatric and adult follow-up models have created gaps in post treatment support for TYAs, and this is largely attributable to the types of cancers and their follow-up pathways, e.g., paediatric type cancers like Leukaemia, require inpatient care while breast cancer or melanoma, which are more common amongst older TYAs are typically treated in outpatient settings [19,20]. In the UK, TYAs (16–24 years) often receive care in specialised units with multidisciplinary support, whereas YAs (25–39 years) are often treated in general adult settings with limited age specific services [21,22]. This transition between paediatric and adult oncology services can create gaps in care, particularly in areas such as psychosocial support, which are not always addressed within adult or adult-focused oncology settings. This highlights a broader need for tailored and integrated follow-up care.

The University Hospital Southampton (UHS) has a dedicated TYA Cancer Unit offering age-appropriate facilities and support for TYAs aged 16–24 years. TYAs receive a clinical end of treatment plan and follow-up care involving multi-disciplinary teams, such as psychologists, youth support workers, social workers and fertility specialists. However, as TYAs fall within the adult model of cancer follow-up, pathways of support can be unclear and inconsistent. Follow up is typically disease-stratified, remote, with limited long-term monitoring of late effects, including psychosocial support needs. This is in contrast with the paediatric model, which typically involves 5 years of disease surveillance followed by risk-stratified review for treatment-related toxicities.

Despite the well-reported long-term impact of cancer on TYAs [3,4,5], little is known about whether current support services adequately meet their needs after treatment-particularly when support is needed, what this support should look like and how it should be delivered. The evidence guiding best practice for TYA follow-up care remains limited [6,23,24]. This current study aims to understand TYAs’ preferences for support post treatment within a UK hospital setting. Uniquely, this study employs a co-design approach, working in partnership with TYAs to identify the key components of optimal supportive care. By prioritising TYA’s preferences and utilising co-design, this study seeks to address the limitations of existing models that may not adequately capture the lived experiences and needs of TYAs. This study contributes to the development of more relevant, acceptable, and feasible solutions within the local healthcare system.

## 2. Methods

### 2.1. Ethical Considerations

Ethical approval for this study was obtained from University of Southampton ethics committee (ERGO 90664 and ERGO 94041). All participants provided informed verbal consent before taking part. The study adhered to ethical guidelines to ensure confidentiality, voluntary participation, and the right to withdraw at any time.

### 2.2. Study Design

The Consolidated Criteria for Reporting Qualitative Research (COREQ) guidelines [25] were followed in reporting the study findings (Supplementary File S1: COREQ guidelines).

This study comprised two phases: Phase 1 involved semi-structured interviews with TYAs who had completed cancer treatment at UHS. The aim of phase 1 was to explore TYAs’ post treatment supportive care experiences, needs and preferences. Phase 2 consisted of co-design workshops with TYAs with a lived experience of cancer and feedback from healthcare/allied professionals (HCAPs), of which the aim was to review, refine and develop support service recommendations.

### 2.3. Participants and Recruitment

#### 2.3.1. Phase 1

Inclusion criteria required participants to be aged between 16 and 25 years at the time of treatment completion and who had completed treatment at UHS between one and six years previously. Participants also needed to be able and willing to provide informed consent.

Eligible participants were identified by the late effects nurse at the participating hospital, from a database of young people in follow up. The registry included people diagnosed within the paediatric or TYA setting but who were TYAs at the time of treatment completion. The late effects nurse contacted potential participants via telephone, providing an overview of the study and emphasising that participation was voluntary.

#### 2.3.2. Phase 2

Co-design workshops were conducted with young people with a lived experience of cancer to review findings from the interviews and collaboratively develop recommendations for improving support services. Members of the University of Southampton’s Young People with a Lived Experience of Cancer Advisory Group, led by SS and NC were invited to participate in this phase if they had been diagnosed with cancer between the ages of 16 and 25 years (no restrictions in terms of treating hospital and treatment status). Participants who took part in phase 1 interviews and expressed interest in taking part in phase 2 were also contacted.

For practical reasons, HCAPs were invited by email to review a summary document (Supplementary File S2: summary document) and a 15 min online presentation of the findings from phase 1, and were asked to provide feedback by email to further refine recommendations developed from TYAs in Phase 2.

### 2.4. Data Collection

Socio-demographic characteristics of TYAs were self-reported and collected before the interviews/workshops.

#### 2.4.1. Phase 1: Interviews with Young People

Semi-structured, in-depth interviews were conducted between June and December 2024. Interviews explored the support participants had received from UHS following the conclusion of their cancer treatment, any additional support they had needed, and their preferences regarding the type, format, and delivery of support services.

Interviews were conducted at a single time point, either via telephone or Microsoft Teams and lasted between 20 and 60 min. The interviews were conducted by NC, a psychosocial oncology researcher experienced in qualitative methods, with a professional interest in the topic. No prior relationships existed between the researcher and participants. The interview topic guide (Supplementary File S3: Topic guide) was pilot tested with a patient partner (PD). Field notes were made to facilitate analysis. Interviews were audio-recorded via Microsoft Teams transcription software and transcribed verbatim. Transcripts were not returned to participants for verification, or correction for both phases.

#### 2.4.2. Phase 2: Co-Design Workshops

The workshops adopted a participatory, co-design approach [26], engaging both TYAs with a lived experience of cancer and HCAPs. NC and CR (a patient partner) co-facilitated the workshops. Each workshop lasted one hour and conducted via Microsoft Teams. The workshop was structured into three main components: reviewing phase 1 interview findings, collaboratively developing practical action points and recommendations, and considering implementation strategies for support services, including different delivery formats (online, face-to-face), and charity-based programmes. The findings of phase 1 were visually displayed via a PowerPoint presentation and explained by the workshop lead (NC). Co-design activity instructions were also presented with an opportunity for TYAs to contribute their ideas on the slides. The first co-design activity invited TYAs to reflect on the themes from the interviews and identify gaps, followed by the creation of a “Dream support package”. Finally, key recommendations were summarised with an invitation for TYAs to vote on their top three or four components of an optimal support system.

Two separate workshops were conducted with TYAs, each lasting one hour, with a maximum of five participants per session. Workshops were audio-recorded via Microsoft Teams transcription software and transcribed verbatim.

Written feedback from HCAPs were analysed and incorporated into the final recommendations.

### 2.5. Data Analysis

For phase 1, Braun and Clarke’s thematic analysis framework [27] was used to analyse the data. This method consists of six phases: becoming familiar with the data, generating initial codes, searching for themes, reviewing themes, defining and naming themes and producing the report. The data were independently coded for five transcripts by two researchers (NC, SS). A primarily deductive approach was used, as coding was guided by predefined areas based on interview questions mentioned above. However, an inductive element was also incorporated to allow for the identification of themes that fell outside of the interview questions. The researchers conducted line by line coding of the interview transcripts, grouping codes with common characteristics into categories, and then refining them into overarching themes. Excel was used to manage the data. The sample size was guided by information power [28]. This approach emphasises the relevance and richness of the data in relation to the study aim, rather than the number of participants. Given the specificity of the research aim, the relevance of the sample, and the depth of data collected, the sample was considered sufficient to support meaningful analysis.

For phase 2, a participatory thematic analysis was used, combining deductive coding aligned with phase 1 themes and inductive coding to capture new insights. Discussions were transcribed, coded iteratively, and refined collaboratively with young people and HCAPs separately, ensuring recommendations were grounded in both research findings and lived experiences.

Discrepancies in interpretation for both phases were resolved through discussion, consensus-building, and reflection on how each code aligned with the data and research aims. This process helped refine the coding frameworks and ensure coherence in theme development across the dataset. Final themes were developed collaboratively, drawing on both researchers’ views.

## 3. Results

A total of 52 participants were invited to take part in phase 1, of which 21 expressed interest; 1 subsequently decided not to participate, and 4 could not be reached. Sixteen interviews were conducted (response rate 30.8%) in phase 1. Fifteen young people with a lived experience of cancer and eleven HCAPS were invited to take part in phase 2 (response rate 50.0%). Eight young people (5 treated at UHS and involved in Phase 1, and 3 members of the Young People with Cancer Advisory Panel) participated in the co-design workshops in phase 2, with feedback from five HCAPs including the TYA lead nurse, social worker, two youth co-ordinators, and late effects nurse. The age range of TYA participants from both phases at study enrolment was 18–30 years and at diagnosis, 16–24 years. Haematological cancers were the most common (75% of phase 1 and 50% phase 2 TYAs). Two of the TYAs in phase 2 were in receipt of maintenance therapy for haematological cancer. Table 1 shows detailed demographic characteristics of TYA participants from both phases.

**Figure 1:**
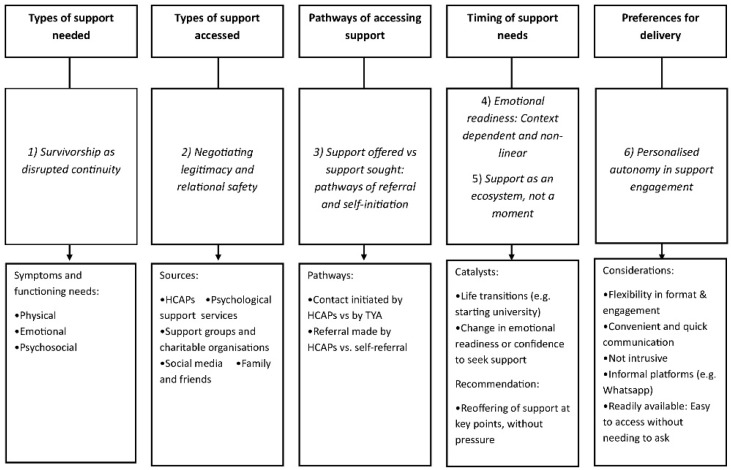
Mapping of support care needs, experiences and preferences from Phase 1 interviews.

Data from phase 1 were categorised into six themes (see Figure 1) covering experiences and preferences within the following questions:

- What support is needed following treatment? *(1) Survivorship as disrupted continuity.*
- What support is accessed by TYAs? *(2) Negotiating legitimacy and relational safety in help seeking.*
- How is support accessed? *(3) Support offered* vs. *support sought: pathways of referral and self-initiation.*
- When is support needed? *(4) Emotional readiness as context dependent and non-linear; (5) Support as an ecosystem, not a moment.*
- What are TYAs’ preferences for support delivery? *(6) Personalised autonomy in support engagement.*

### 3.1. Phase 1 Semi-Structured Interviews

#### 3.1.1. What Support Is Needed by TYAs Following Treatment?

**(1)**  **Survivorship as disrupted continuity**

TYAs accounts of their support needs post treatment reflected experiences of disrupted continuity; a sense that for some, life, body and a sense of self have not returned to ‘normal’. Support was sought across physical, emotional and psychosocial domains.

*Physical*

TYAs reported accessing support to manage new or ongoing symptoms after treatment. One participant described his experience of seeking support when new symptoms appeared: *“[…] because my tonsils had grew two and half times the size of what they should have been. And because they’re made of the same material as the lymph nodes, they [nurses] instantly flagged it up and …had it removed* (SMY021: male, 20 years). Another participant sought support regarding post-surgery health concerns, stating, *“Post surgery like forever since, I’ve never felt right and I don’t know whether it’s anything to do with my surgery or whatever”* (SMY010: male, 25 years).

*Psychosocial*

Anxiety, trauma, and the emotional challenges of reintegrating into society were significant concerns for some TYAs following treatment. The psychological impact of illness was also apparent, with one participant expressing the ongoing anxiety related to their health: *“I have to constantly remind myself that I’m not gonna die from a cold and it’s okay whenever it happens”* (SMY001: female, 24 years). Body image and social anxiety were further concerns: *“I found it really hard. I was embarrassed about my hair, for instance. I found that really hard. A lot of my like chats with the psychologists were about like… like the social anxiety around it”* (SMY002: female, 25 years). Life transitions, such as returning to university, also drove the need for support. One participant noted the challenge of adjusting to university life after treatment. Grief was another factor that led to seeking social support. A participant reflected on the loss of a family member to cancer, stating, *“I could have been in that position as well… it sort of caught up with me”* (SMY021: male, 20 years).

#### 3.1.2. What Support Is Accessed by TYAs?

**(2)**  **Negotiating legitimacy and relational safety in help seeking**

TYAs navigated support from formal and informal sources, but their access was influenced by their sense of legitimacy (i.e., whether certain services/support was ‘for them’) and whether particular relationships of services felt emotionally safe or approachable enough to engage with. These negotiations shaped how, when and from whom they sought help. TYAs accessed support post-treatment from sources including HCPs, youth worker support, social worker support, psychological support services, social media, support groups, family, friends, and partners, and charities.

*HCAPs*

Members of the clinical team, in particular nurses, were described as only being a phone call away in the event of accessing advice for medical concerns. HCAPs were generally praised for their responsiveness and care, with one participant noting: “*they give me contact details for people at the hospital that I can speak to if I have any issues […] I mean, all of the all of the nurses were so amazing. So I felt like you could talk to them about things and they’d always point you in the right direction if you needed anything.*” (SMY014: female 24 years:).Though, some felt disconnected or initially intimidated, saying, “*So they can be quite intimidating […]. You had to really gear yourself up to say something… and there have been the odd few times where I’ve walked away being really frustrated and upset. […] they’re the reason I’m alive, and I’d much rather like someone be blunt and straight with me*” (SMY01: female 24 years).

Youth worker support provided an important emotional outlet, with one participant expressing, “*I would just moan at them or not at them but with them about how it just felt quite unfair [being diagnosed with cancer] yeah, and because they’re not therapists in any way, they’ve heard this quite a few times and they kind of normalized it which was good. But I like that they don’t pretend to be something they’re not.”* (SMY007: female, 18 years). Social workers, offering both emotional and practical assistance, were appreciated by some: “*Even after treatment, they would message me, or even I would message them […] asking me to go on trips and stuff … or afternoon tea. So that’s always nice to have someone actually still there that you can message*” (SMY006: male, 18 years). Another participant shared, *“If I hadn’t have had that social worker at the time I probably wouldn’t have known some of the funding that you can […] get for different things”*(SMY015: female, 22 years). However, some participants expressed disappointment when follow-up communication from HCAPs was less consistent, with one noting, *“I haven’t heard from her in almost a year”* (SMY007: female, 18 years).

*Psychological support services*

Psychological support was helpful for many, such as one participant who shared, “*[…] Therapy really helped me understand everything properly… it was sort of like grieving, so then the therapy really helped me become me again*” (SMY020: female, 25 years), although others found it less beneficial, stating, “*It wasn’t for me… I didn’t want to constantly think about it*” (SMY018: male, 19 years).

*Support groups and charities*

Sharing experiences with other TYAs who had walked a similar path was valuable for some, and favoured over formal counselling: “*And I think there were things that you would never be able to talk to anyone else about or what no one else would ever understand. […] So although I didn’t necessarily feel like I needed like counselling or anything, I did want to keep talking about it and I think that was quite healthy for me.”* (SMY008: female: 24 years). As well as offering a platform to connect with others, charities were also a significant source of practical help, particularly for financial and material support.

*Social media*

Social media had mixed outcomes; some found it supportive, with one participant commenting on the use of Instagram, “*I did find it on Instagram, but like lots of kind of people that were talking about kind of what they kind of achieved post treatment, I found that was really helpful for me.*” (SMY015: female, 22 years). While others talked about the negative psychological impact of exposure to conversations on such forums “*I joined a Facebook group about people who had cancer… and I found that they shared really morbid stories, and sometimes that just doesn’t help someone who’s trying to recover from their own story*” (SMY020: female, 25 years).

*Family and friends*

TYAs also described family, friends, and partners as the backbone of support: “*My mum deals with it all… basically my friends and family are my support system*” (SMY016: female, 20 years), while another shared, “*My partner is such, like he’s my rock… I can speak to him about anything…*” (SMY020: female, 25 years).

#### 3.1.3. How Is Support Accessed?

**(3)**  **Support offered vs. support sought: pathways of referral and self-initiation**

TYAs accessed support through different pathways, either accepting support that was offered to them and/or actively seeking help on their own. Some appreciated the proactive offers from healthcare teams, while others chose to seek help independently when they felt ready. When seeking psychological support, many TYAs were referred to specific types of services by HCAPs, such as psychologists or counsellors. One participant recalled, “*I had a whole meltdown and said to my mum, I don’t wanna do it anymore, […], I can’t do it… so she went and spoke to one of the nurses who came and had a chat with me and referred me like internally to the psychologist*” (SMY001: female 24 years). In this case, the psychological support was provided by a hospital-based psychologist. Others were directly offered psychological support by their clinical teams, including referrals to talking therapies or counselling. For example, one participant mentioned, “*The hospital (clinical team) probably offered it. I definitely didn’t ask for anything*”(SMY017: male, 23 years).

The availability and proactive nature of these services were frequently highlighted as important factors in accessing support. One participant reported, “*It was good they [*consultant*] were quite proactive with it… I never thought of actually, maybe I should see someone for this. And my consultant, she spoke to me […] she gave me a bit of a talk about how there’s nothing wrong if you want to seek help and we all would support you in that, if that’s what you need.*” (SMY007: female, 18 years), referring to being offered a referral to therapy by their healthcare team. In terms of support groups, TYAs were referred to peer support networks/groups by either their social worker or youth coordinator: “*It was definitely my social worker, that told me about it and she would just kind of text me things and be like, feel free to reply if you’re interested and stuff like that […]*” (SMY008: female, 24 years). Conversely, participants described having to actively seek out and self-refer to formal therapy support services post treatment, while another sought private therapy independently two years post-treatment.

#### 3.1.4. When Is Support Needed?

**(4)**  **Emotional readiness as context dependent and non-linear**

Emotional readiness to seek help did not follow a predictable trajectory. TYAs sought support at various points throughout the post treatment period, with the timing often influenced by their life transitions, emotional processing, external triggers and evolving needs. For example, as mentioned above with the transition to university or with a family bereavement acted as catalysts for seeking support. HCPs were frequently contacted at different stages, particularly for physical concerns or ongoing management. One participant reflected on how their approach to healthcare changed over time: “*I think especially at the point where I’m at now, at the beginning I would have asked a lot more questions. But now I’m somewhat in a place where I want to just get on with my life and I don’t*” (SMY001: female, 24 years). In contrast, another participant reported a shift in their confidence in contacting the healthcare team, saying, *“I’ve become so much more confident. I wouldn’t have accepted doing this call if I hadn’t gone through everything”* (SMY016: female, 20 years), highlighting a growing self-assurance in engaging with HCPs.

Psychological support was sought at various stages of treatment and post-treatment. Some TYAs initially declined psychological help but later sought it when they recognised their emotional needs. One participant explained how they turned down further psychological support during treatment, stating, *“I think I took that slightly as my get out of jail free card and I was like I’m totally fine, I’m so fine, we don’t need to do this anymore”* (SMY001: female, 24 years). This participant later acknowledged that while the support was helpful, they were not ready to properly address their emotional struggles at that time. Another participant reflected that, while support was available, they hadn’t initially recognised the need for psychological help, stating, *“I did feel quite rubbish, but I pin that as, oh, that’s normal with what you’ve gone through. And I never thought of actually, maybe I should see someone for this”* (SMY007: female, 18 years). In another case, counselling was sought after treatment had ended, with the participant indicating they were more open to it once they felt ready: *“I said, that’s not something I want till after treatment. And then as soon as treatment ended, she [social worker], had remembered and followed me up on it”* (SMY008: female, 24 years).

Social support was often sought later in the recovery process. One participant described how they initiated contact with a social worker after treatment to stay updated and manage their recovery, saying, *“It was more me. Normally, I’m not the best person at initiating anything… but then I thought they were there and they’re in that job for a reason. So, I just went for it really”* (SMY006: male, 18 years). Similarly, peer support was sought several months after treatment. One participant shared, *“I’ve gone to a few [social events/workshops with other TYAs] but probably my first one I went to was about nine months post-treatment”* (SMY007: female, 18 years).

**(5)**  **Support as an ecosystem, not a moment**

TYAs reported that suitable support must evolve and be sustained, rather than delivered as a one-time offer. Many TYAs believe psychological support should be offered early, ideally before or during treatment. However, for others, support should also be reoffered later, particularly after the first year post-treatment when patients are adjusting to life again; *“It took me like a good year after my transplant for me to even approach the idea that I might need to sit down [ask for help] and then I wasn’t ready at that point. I knew I needed to do it, but I wasn’t ready to do it. It wasn’t until like two and a half years after my transplant that I actually was like, okay, no, I need to do this”* (SMY001: female, 24 years). The majority suggested reoffering support during follow-up appointments, with many preferring a non-pressuring approach, such as, *“Mention it loosely, like, it’s still here if you need it. Like, just, just give me a text.”* (SMY007: female, 18 years). Some expressed discomfort with repeatedly being offered therapy, feeling it could be overwhelming or unwanted; *“[…] Some people think therapy is the only option… so like when you know therapy is not really your thing, you don’t want to be constantly offered it”* (SMY018: male, 19 years). Additionally, participants emphasised the value of informal check-ins during routine appointments, with one suggesting, *“I think even if it’s something that’s just mentioned in your regular checkups, […] if you feel you’re fine, then don’t worry. But as a standard, mention to patients around this time about this, just like a general check-in*” (SMY013: female, 30 years), reflecting a preference for a flexible and supportive approach.

#### 3.1.5. How Do TYAs Prefer Support to Be Delivered?

**(6)**  **Personalised autonomy in support engagement**

TYAs expressed clear preferences for different formats of support depending on the type of service-options that allow them to retain autonomy over how, when and if they engage with support. For communication with HCAPs, the most preferred format was text messaging, as it was seen as convenient and less intrusive. One participant highlighted, *“They were really good to just be able to text, which was really helpful because you always want to have a really informal conversation. It was just a quick text to the TYA team.”* (SMY008: female, 24 years). However, some participants still preferred phone calls for urgent issues, with one participant stating, *“I prefer to call them […]. Just so I know they’ve received the message, even if it is a voicemail.”* (SMY021: male, 20 years). For psychological support, many participants desired an opt-out model rather than the responsibility of initiating the request themselves. As one participant noted, *“If it were more of a everybody gets it and then you can opt out of it rather than you can opt in because a lot of the time the hardest part is actually telling someone saying I need this […]?”* (SMY001: female, 24 years). In terms of youth worker and social worker support, TYAs preferred WhatsApp for its informal, accessible, and convenient nature: *“WhatsApp. So it was quite accessible and easy rather than just sending an email or having to call them. I’m normally quite bad when someone calls me and it’s a number I don’t recognize.”* (SMY006: male, 18 years). Despite this, charity-based support such as leaflets and in-person visits were not as well-received, with one participant saying, *“If I get too many things handed to me, I wouldn’t know where to go”* (SMY020: female, 25 years), and another expressing that visiting the hospital-based cancer support service (Macmillan Cancer Centre) left them feeling sadder rather than supported. For support groups, participants preferred both online and in-person formats. Finally, social media, particularly Facebook groups, was recognised as useful for sharing information, including financial support details. Overall, TYAs preferred flexible, accessible, and informal communication methods across different types of support, with a focus on convenience and personalised care.

### 3.2. Phase 2 Co-Design Workshops

The feedback from the content of phase 1 interviews and participants’ “Dream Support Package” generated the following recommendations from TYAs to enhance post-treatment support.

#### 3.2.1. Clear Pathways for Long-Term Support

Confusion over what was on offer and by whom was communicated especially when care was delivered across different teams: “*So a lot of stuff got missed because TYA thought Gynae were doing stuff……I should have been entitled to more support…if it was more clear cut then things wouldn’t have been missed”* (SMYWS001: female, 20 years). This also linked with the desire for continuity of care over time by a dedicated long-term follow-up service delivered by people who were familiar: *“you don’t really want to have to go through the GP or anything because that’s just a long route for something that you really should be able to ask”* (SMYWS002: female, 30 years). One TYA mentioned that her follow-up care was transferred to a different hospital when she relocated for university: *“Like my CNS [Cancer nurse specialist] that I’ve got at Uni [University], she’s lovely but I can’t text her. And she is also a bit older, so it’s harder to relate”* (SMYWK003: female, 24 years). Conversations with the nursing team at the end of treatment about what to expect in terms of support on offer and the period covered was also recommended.

#### 3.2.2. Flexible and Periodic Check-Ins

Scheduled regular check-ins were recommended following treatment but also with flexibility to coincide with critical life milestones, for example, when planning to start a family, or leaving the family home to start University. Regular check-ins also serve as a safety mechanism with the provision of support for long-term and late physical and psychological effects of treatment, and reassurance that no signs of recurrence are missed. In terms of when support should end, it was recognised that support might be needed several years post-treatment: “*I think there’s no like end point for when you’re going to have, like questions like, we’re quite young if in like 5–10 years time we’re thinking about like having children and kind of probably going to want to talk to somebody about like where*”*….*“*I don’t want to have those conversations now because I have no idea what I need”* (SMYWK004: female, 18 years).

#### 3.2.3. Access to Peer Support and Confidence-Building Activities

Opportunities to meet with other TYAs with shared experiences in a relaxed environment was recommended. TYAs reflected on events and activities they had engaged in and acknowledged that not all activities would be suited to all TYAs: “*it’s about finding the right thing…I got invited to this boat trip which wasn’t really my type of thing*” (SMYWS005: male, 18 years). Recommendations were made for both in person adventurous and confidence-building activities (e.g., outdoor events, running club/event, leadership challenges) at regular intervals to foster social interaction and personal growth as well as virtual support forums.

#### 3.2.4. Long-Term Digital and App-Based Support

The use of digital technologies was proposed to support the long-term needs of TYAs, with one workshop identifying the use of apps, in particular to contact HCAPs for further support when it is needed the most as the top priority for a “dream support package”: *“So actually having that app where there’s like, I’m not going to say 24/7 help, but if you could just write a message on and then someone to respond to you*.” (SMYWS005: male, 18 years). “*In the perfect world, apps will be available for us to ….All this information would be easy to access and it’s more likely that people actually respond to it.”* (SMYWK006: male, 29 years). It was proposed that apps could also include emergency contact features as well as clear guidance on who to contact for specific needs such as, clinical, emotional, practical, or life-planning. Periodic updates via digital platforms could also serve as a reminder of available services. It was also recommended that email or digital check-ins allow TYAs to reflect and respond at their own pace, although if these were unexpected, they could be “triggering”.

#### 3.2.5. Emotional and Psychological Wellbeing Support

Addressing psychological and emotional support needs was recognised as an area requiring particular attention with one TYA describing a gap in provision and uncertainty as to where to access help post treatment: *“The bad days, just like who do I contact?”* (SMYWS005: male, 18 years). Regular mental health check-ins through digital platforms were favoured, where individuals can respond to questionnaires, track their emotional health, and access help as needed. This should allow flexibility and give individuals time to reflect on their feelings without the pressure of phone calls. In addition, the provision of informal support (not necessarily led by mental health practitioners) offering TYAs a space to discuss their fears and concerns.

#### 3.2.6. HCAPs’ Review of Recommendations

HCAPs acknowledged that the pathways of follow-up for TYAs are not clear given that their follow-up is specific to their cancer-type and that there are no late effects clinic for TYAs. There was a shared vision for flexible, continued holistic (not just medical) support. Even though the youth support workers provide support to TYAs for up to 2 years post-treatment, they reassure TYAs that they are welcome to contact them for support irrespective of the time since treatment completion. Follow-up events had been organised for TYAs in the past although the uptake was low. There is a lack of confidence amongst the HCAPs that they are successfully reaching TYAs; newsletters are emailed to them signposting to events and charities, but it was acknowledged that TYAs do not regularly check their emails. The youth support worker described not being able to keep pace with the ever-changing communication channels used by TYAs although they were always *“thinking outside of the box”* to fit into where they are needed.

## 4. Discussion

This study explored the support needs of TYAs with a lived experience of cancer, and found that TYAs experience wide ranging, complex and evolving support needs long after cancer treatment ends, particularly around physical, psychological wellbeing, social reintegration and life milestones. Support needs are highly variable and often do not surface immediately after treatment; instead, many TYAs prefer to *find their own way* initially, relying primarily on informal support from family and friends rather than formal services. Current follow-up care is often reactive, inconsistent and not always well timed to meet TYAs emerging needs. Many TYAs accessed support only when reaching a point of crisis, highlighting a gap in proactive support and the communication of available ongoing service provision. HCAPs and peer support were valued by participants. Consistent with previous research, cancer-peer connections play an important role in AYA cancer care during treatment and survivorship, offering emotional reassurance, reducing distress and enhancing cancer related knowledge [29]. The literature also echoes our findings that hearing about negative experiences from peers can also trigger anxiety or distress. This highlights the need to strengthen and personalise peer support pathways to ensure they are safe, supportive and responsive to individual preferences.

Another key finding was the *timing* of accessing support, with some TYAs referring to the need to be ‘ready’ to access support. The need for support often became more apparent, around significant life events such as starting university. This delayed engagement suggests that TYAs may need time to adjust to life after treatment before seeking emotional and social support from peers or professionals. This contrasts with existing literature, which identified barriers to seeking help, such as embarrassment, a belief that their concerns were normal or perceptions that nothing can be done [23]. Our findings suggest that while these barriers may exist, for many TYAs, it is emotional readiness and personal timing, rather than stigma alone that governs help seeking behaviour. Participants valued the idea of support being *reoffered* over time, recognising that specific needs become more focal at different stages of life and that individuals follow different trajectories. This approach is supported by evidence that early and repeated supportive care interventions can help to reduce the long-term impact of side effects [30,31]. Casual, informal interactions were also seen as valuable, suggesting that support does not always have to be delivered through formal therapy pathways. Further consideration should be given regarding who is best placed to provide this support, as it may not always need to come from HCPs; community based, or informal networks could also play a key role in meeting the diverse needs of TYAs.

TYAs clearly expressed the need for long-term, flexible, and easily accessible support options, in particular psychological check in’s that are reoffered over time and informal, digital modes of communication. This aligns with previous research identifying significant gaps in follow-up care for young adult cancer survivors, including poor preparation for survivorship, lack of interdisciplinary support, and unclear care coordination [32]. Our findings further support the importance of personalised approaches tailored to different life stages, acknowledging that individuals vary in their emotional readiness to seek help [31]. Our co-design work also recommended the need for continued support for TYAs, accessible through multiple routes including informal, peer-led and digital formats designed to respect personal readiness and individual differences.

Although HCAPs recognised the importance of holistic, long-term support, they also highlighted significant barriers to delivering it. Pathways of care were often fragmented with follow up tied to cancer type rather than survivorship needs. In addition, traditional communication methods, such as email, were poorly aligned with how TYAs prefer to engage. These challenges echo existing concerns about the lack of coherent survivorship pathways for this group [10,33]. Although the recommendations from the study align with clinical aspirations, feasibility is currently limited by systemic constraints, resource challenges and the need for more innovative youth centred models of follow-up care that support evolving fast paced technological advancements (e.g., social media platforms, artificial intelligence, i.e., chatbots). The challenges of coordinating cancer survivorship care are not unique to TYAs; a review of the implementation of the landmark Institute of Medicine Recommendations for cancer survivorship revealed persistent gaps in care delivery/ provision, with patients still “lost in transition” [34,35]. Policy development such as the Children and Young People’s 10 Year Cancer Plan outline important national priorities aimed at addressing these issues [36]. These include the implementation of a national survivorship passport scheme, evidence-based surveillance guidelines, provision of long-term psychosocial support and the establishment of a joined up single point of access for long-term follow-up care. Our work contributes to how such policies might operationalise some of these commitments in practice, ensuring they are both aligned with the lived experiences and preferences of TYAs.

The study has several strengths, including its co-design participatory approach and the inclusion of two patient partners (CR and PD) with lived experience of cancer, who contributed to the design and delivery of the study. Close collaboration with the clinical team further strengthened the study’s practical relevance. However, there are limitations to acknowledge, including the lack of diversity of the TYAs involved in this study. Potential participants were screened by their clinical team, and it is likely that we have missed TYAs who are “seldom asked”. Lack of representation is a commonly reported challenge in the literature [37,38]. This selection bias may have particularly excluded TYAs with limited internet access or digital access barriers. Recruitment issues and a low response rate of TYAs, as well as lack of involvement of senior HCAPs, and policy makers limits the breadth of perspectives captured. For logistical reasons, HCAPs reviewed a report detailing the recommendations and provided written feedback to the research team. Organising a follow-up workshop bringing together TYAs and HCAPs could have offered more in-depth insight into the TYAs needs and preferences as well as the barriers to survivorship care. Challenges we faced in terms of “reaching” TYAs were also shared with HCAPs who expressed concerns about the limited success of their current efforts. TYAs involved in our earlier research recommended that “we meet them where they are at” [39]. Finally, as this study was conducted at a single UK hospital, the transferability of findings to other regions or healthcare systems is limited and should be interpreted as relevant. Continued engagement with TYAs to better understand their needs and preferences will help ensure that the support on offer matches what they need, when they need it and how they would like to receive it. Our work represents an important step in this direction, offering clear co-designed recommendations. However, meaningful progress will require broader systemic efforts, particularly within resource-limited settings to translate these findings into sustainable, equitable support pathways.

## 5. Conclusions

TYAs experience diverse, often complex, support needs following treatment for cancer, demanding a personalised, flexible approach. Support must be proactively reoffered at key life stages, using informal and accessible communication methods that fit with TYAs’ preferences. Recognising that not all TYAs will engage with every form of support; support services should offer a range of options, allowing TYAs to choose what feels right for them at different times. While HCAPs support this vision, fragmented pathways and practical challenges limit current delivery. Coordinated survivorship care should consider including support systems that respect personal readiness and preferences by providing timely check ins, and adaptable peer support options tailored to individual interests and including confidence building activities. Support must be accessible through a variety of channels, such as community programmes, digital platforms or direct contact with HCAPs, to ensure TYAs can connect in ways that suit them. Clear, ongoing communication strategies are essential to ensure TYAs are aware of what support is available and how to access it. Future research should continue to work in partnership with TYAs to develop and evaluate innovative models of support that can flexibly meet the needs of this population over time.

## Figures and Tables

**Table 1:** Characteristics of participants in Phase 1 and 2.

Characteristics	Phase 1	Phase 2
	Number (%)	
**Age at diagnosis**		
16–18	10 (62.5)	4 (50.0)
19–21	3 (18.8)	2 (25.0)
22–25	3 (18.8)	2 (25.0)
**Age at interview**		
18–19	4 (25.0)	2 (25.0)
20–22	4 (25.0)	2 (25.0)
23–25	7 (43.8)	2 (25.0)
26–28	0 (0.0)	0 (0.0)
29–30	1 (6.3)	2 (25.0)
**Sex**		
Female	9 (56.2)	6 (75.0)
Male	7 (43.8)	2 (25.0)
**Ethnic Group**		
White	16 (100.0)	7 (87.5)
Bangladeshi	0 (0.0)	1 (12.5)
**Nationality**		
British	16 (100.0)	8 (100.0)
**Type of cancer**		
Lymphoma (Non Hodgkin and Hodgkin)	9 (56.2)	4 (50.0)
Leukaemia (ALL)	3 (18.8)	1 (12.5)
Brain tumour	1 (6.3)	1 (12.5)
Bone cancer	1 (6.3)	0 (0.0)
Thyroid cancer	1 (6.3)	0 (0.0)
Cervical cancer	1 (6.3)	1 (12.5)
Myeloproliferative neoplasm	0 (0.0)	1 (12.5)
**Years since completion of treatment**		
1 year	5 (31.2)	2 (25.0)
2 years	2 (12.5)	0 (0.0)
3 years	6 (37.5)	0 (0.0)
4 years	2 (12.5)	1 (12.5)
5 years	0 (0)	0 (0.0)
6 years	1 (6.3)	1 (12.5)
Not known	0 (0.0)	4 (50.0)
**Employment**		
Part-time	5 (31.2)	3 (37.5)
Full time	8 (50.0)	2 (25.0)
Student	1 (6.3)	3 (37.5)
Not working	2 (12.5)	0 (0.0)
**Living arrangements**		
Living with parent(s)	12 (75.0)	6 (75.0)
Living with partner	3 (18.8)	1 (12.5)
Living with friend	1 (6.3)	0 (0.0)
Living alone	0 (0.0)	1 (12.5)
**Education level**		
Secondary education (GCSE’s)	3 (18.8)	1 (12.5)
Further education (A Level, BTech, NVQ)	6 (37.5)	2 (25.0)
Higher education (undergraduate)	6 (37.5)	4 (50.0)
Higher education (postgraduate)	1 (6.3)	0 (0.0)
Not known	0 (0.0)	1 (12.5)

## Data Availability

For further inquiries regarding the data supporting the conclusions of this study, please contact the corresponding author.

## Supplementary Materials

The following supporting information can be downloaded at: https://www.mdpi.com/article/10.3390/curroncol32060361/s1, Supplementary File S1 COREQ checklist; Supplementary File S2. Summary of findings; Supplementary File S3. Topic Guide.
