# Supplementary material for: SUPPORT MY WAY: Supporting Young People After Treatment for Cancer: What Is Needed, When This Is Needed and How This Can Be Best Delivered
Source: Curr Oncol. 2025 Jun 19;32(6):361. doi: 10.3390/curroncol32060361 (PMC12191873; doi:10.3390/curroncol32060361)
Supplement: Supplementary file 1 [file curroncol-32-00361-s001.zip › Supplementary File 2. Summary of findings.pdf]

SUPPORT MY WAY

SUPPORTING YOUNG PEOPLE AFTER TREATMENT FOR CANCER: WHAT IS NEEDED, WHEN IS THIS NEEDED AND HOW CAN THIS BE BEST DELIVERED.

Teenagers and young adults (TYAs) who have undergone cancer treatment often face challenges transitioning to life after treatment, experiencing anxiety about recurrence, long-term side effects, financial instability, and fertility concerns. The University Hospital Southampton (UHS) has a dedicated TYA Cancer Unit offering age-appropriate facilities and support for TYAs aged 16-24 years. TYAs receive a clinical end of treatment plan and follow-up care involving multi-disciplinary teams, such as psychologists, youth support workers, social workers and fertility specialists. However, gaps remain in extended monitoring for late effects, psychosocial support, and the transition from paediatric to adult follow-up care. UHS patient feedback suggests that support could be improved after treatment.

Project Aims

- **Phase 1**: To assess support provided to TYAs (aged 16-25 years when treatment for cancer finished and who completed treatment between 1 and 6 years ago) at UHS to explore their experience of support, identify areas where they require support, how they prefer to receive this support, and when they need it.
- **Phase 2**: To develop recommendations to improve post-treatment support.

Research Findings

- **Interviews**: Conducted with TYAs (n=16) via Microsoft Teams.
- Average age of participants at diagnosis was 18.8 years.
- **Workshops**: Held with young people (n=8) and healthcare professionals (n=5).

Types of support accessed post treatment

- **Healthcare professionals**: Nurses, oncologists.
- **Social & charitable**: Social workers, youth workers, cancer charities, support groups.
- **Psychological**: Counsellors, psychologists.
- **Family & family**
- **Other**: Social media, faith communities.

Reasons for seeking support

- **Practical & medical-** treatment related, financial, egg freezing
- **Emotional & psychological-** anxiety management, social integration, coping with isolation, life changes

- **General support-** peer to peer support, spiritual & religious

Experiences of support

- **Healthcare professionals**: Reassured by nurses' hotline, youth workers and ongoing social worker contact which is personalised to the individual.
- **Social media**: Mixed experiences with social media; supportive communities, but also negative impacts from morbid stories.
- **Psychological support**: Varying readiness to engage with therapy, some positive experiences, talking therapies aren't right for everyone; other ways to process experiences should be acknowledged/offered, with ongoing access for this type of support.
- **Peer connections**: Importance of TYA meetups for normalising experiences and providing peer support.

Access to support and timing

- Mostly through healthcare professionals, social workers, self-referral or family.
- People seeking mostly psychological support up to 5 year post treatment.
- Important that psychological support is re-offered at certain time points post treatment.
- Support needed was prompted by major life events and transitions.

Delivery of support

- Text: Convenient, quick, and informal.
- Phone calls: Preferred for urgent or complex issues.
- Face-to-face: Important for counselling or deeper discussions with HCPs, but less comfortable with family present.
- Online options: Provide privacy, flexibility, and connect participants with peers and resources

Recommendations:

Ongoing medical and psychological support

- Clear guidance and pathways for care, ensuring **young people know which professionals (CNS, therapist, GP, social worker) to contact for specific issues** (e.g., physical symptoms, mental health, fertility concerns).
- **Milestone check-ins** at 6 months, 1 year, 5 years, and 10 years, beyond standard annual reviews, to proactively address evolving medical, psychological, and lifestyle concerns.
- **Flexibility in accessing support**, allowing individuals to request more frequent check-ins if needed.
- A **dedicated space to submit questions and concerns to a nurse or appropriate professional** who can respond or refer accordingly.
- **Informal mental health support**, allowing for **casual chats** rather than only structured symptom-based check-ins.

Digital and remote support

- **Digital and app-based support** for guidance and self-help options **when in-person care isn't immediately needed.**

- **Signposting to future services**, such as fertility advice, career guidance, and mental health resources, as life changes.

Peer and social support

- Peer support networks, including **informal meetups** and structured **survivorship workshops**.
- **Age-appropriate** social gatherings, ensuring teens and young adults have **tailored spaces for connection.**
- Access to **activities** that **help with confidence-building** post-treatment.

"I think there's no like end point for when you're going to have questions, like, we're quite young. If in like 5-10 years time we're thinking about like having children and kind of probably going to want to talk to somebody about like if there's anything that I need to be thinking about?Are there other things that I'm going to need to do? But I don't want to have those conversations now because I have no idea what I want." (ID005- quote from young person)
