# Supplementary material for: SUPPORT MY WAY: Supporting Young People After Treatment for Cancer: What Is Needed, When This Is Needed and How This Can Be Best Delivered
Source: Curr Oncol. 2025 Jun 19;32(6):361. doi: 10.3390/curroncol32060361 (PMC12191873; doi:10.3390/curroncol32060361)
Supplement: Supplementary file 1 [file curroncol-32-00361-s001.zip › Supplementary File 3. Topic Guide.pdf]

Interview schedule: Support My Way

This interview schedule is intended as a guide and offers broad questions to be covered along with suggested ways of asking. The exact way questions are asked may differ in order to explore and probe the individuals' experience.

Consent

1. Information sheet – check they have read it and understand it
2. Reinforce: you can ask me to stop at any time, confidentiality

Introduction

We want to talk about the support you received at UHS, any supportive services or resources you have used during treatment or since you completed treatment, your preference in the type of support needed, the way in which it is delivered and the timing of when it's needed. If you have not used any supportive services or resources, we will talk about your views and why that is.

Background information

- o Sociodemographic details: age, employment status, marital status, living arrangements, ethnicity, highest educational qualification
- Medical characteristics: type of cancer, date of diagnosis, treatment received, date of end of treatment, any adjuvant treatment(s).

- **Could you tell me about the experience you have had with the hospital during treatment and since your treatment finished?**
  - o Ask for specific examples of positive impact or challenges
- Evaluation of late effects service- what was your experience like of the support received, how often was contact made, what support was offered, evaluate Ready Steady Go and Worry thermometer.

- Details of the supportive service/resource
    - Description of what the supportive service/resource was
- Deciding to seek support
    - What issue was it that made you decide to seek help from that supportive service/resource
    - Why decided to use that particular service/resource
    - When was contact with the supportive service/resource made?
- Finding out where to access supportive service/resource:
    - What hoped to get from it?
    - How did you go about finding out about it?
- Making contact
- Using the supportive service/resource
    - How did that go?
- Evaluating the supportive service/resource
    - How did you find using the supportive service/resource?
    - Did you get what you hoped from it?
    - What were the challenges in accessing this supportive service/resource?
    - Is it ongoing, or when and why finished?
- Did you have any problems or issues for which you did not seek help or support?
    - Would you have liked any help or support for this problem or issue?
    - If so, what stopped you from finding help or support?
    - What might have helped you to seek help or support?
    - How did you feel about seeking help and support for this issue?

<u>Evaluation in relation to NHS care</u>

- *Contact with specialist*
- *Contact with GP- have they felt heard and understood at GP?*
- *Contact with any other NHS services*

IF THE INTERVIEWEE HAS NOT USED ANY SUPPORTIVE SERVICES/RESOURCES, talk through any problems or issues they have reported to the interviewer, asking:

- Would you have liked any help or support for this problem or issue?
- Did you try to find any help or support for this problem; how did you go about this?
- What happened?
- What stopped you from finding help or support?
- What might have helped you to seek help or support?
- How do you feel about seeking help and support for this issue?

Evaluation of support overall

- Was there anything that you would have liked (more) support with but didn't get? Why not?
- areas where you required support
- how you prefer to receive this support (online, face-to-face, through charities, etc.)
- and when did you need this support?

Ending the interview

- Is there anything else that I haven't asked you about in relation to supportive services/resources which is important?

Interview close

- Give thanks
- what happens now
- any questions
